# Ethical issues under the pressure of COVID-19 pandemic

**DOI:** 10.1192/j.eurpsy.2021.46

**Published:** 2021-08-13

**Authors:** B. Carpiniello

**Affiliations:** Italian Psychiatric Association; Secretary of The Epa-council of Npas, Department of Medical Sciences and Public Health University of Cagliari, Cagliari, Italy

## Abstract

The pandemic has highlighted with particular evidence the vulnerability of people with mental disorders and a series of specific ethical concerns regarding their condition. First of all, the risk of receiving poor medical care due to the double stigma of being affected by a mental disorder and Covid infection, in addition to the many other additional barriers, including poverty, marginal housing, and food insecurity. Moreover, in some countries, in a situation where demands for intensive care exceeded the treatment facilities available, the tragical ethical dilemma regarding the choice of people to be saved was resolved with the option in favor of healthier and/or younger people who have more chances of recovery, thus excluding, among others, aged people with severe mental disorders such as dementias. In other countries, ethical concerns emerged related to the enhanced risk of involuntary hospital admission of individuals with severe mental illness mainly due the high likelihood of these patients violating physical-distancing and other safety rules. Social distancing measures have determined, among others, relevant obstacles for direct access to psychiatric care services, with the consequent adoption of the so called “telepsychiatry” of “tele mental health” by mental health services, a measure which unfortunately has cut off a large amount of patients who have not been able to benefit from these innovative methods of care both because of barriers posed by their own serious mental conditions, and by the impossibility of having the necessary technology.

**Disclosure:**

No significant relationships.

